# A novel integrative multi-scale framework of inflammation and mechanical loading in knee osteoarthritis

**DOI:** 10.1007/s10237-026-02072-8

**Published:** 2026-06-03

**Authors:** Juntong Lai, Damien Lacroix

**Affiliations:** 1 Insigneo Institute, University of Sheffield, Sheffield, UK; 2 School of Mechanical, Aerospace and Civil Engineering, University of Sheffield, Sheffield, UK

**Keywords:** Multi-scale model, Obesity, Knee joint, Inflammation, Cartilage degeneration

## Abstract

Obesity is a well-known prominent risk factor for knee osteoarthritis (OA). The onset and development of knee OA are affected by multifactorial interplays, involving the degeneration of articular cartilage. Emerging evidence shows the negative effects of obesity on cartilage degeneration across multi-scales. Specifically, obesity can stimulate inflammation and alter the biomechanical responses of the joint. However, the pathology of obesity-associated knee OA is still poorly understood. The aim of this study was to develop a multi-scale modelling framework to simulate and evaluate the mechanobiological roles of obesity in the degenerative process of cartilage. This framework integrated the inflammatory and biomechanical effects of obesity on cartilage degeneration in knee OA. A validated finite element model of a subject-specific knee joint was coupled with a mathematical model of adipokine-mediated OA inflammation. In the algorithm, excess stress resulted in mechanical damage that activated obesity-related inflammatory responses. The degeneration of cartilage was driven by both mechanical damage and body mass index (BMI). Parameter sensitivity analysis showed a good adaptivity of this framework to simulate cartilage degeneration. In addition, BMI and the stress threshold were sensitive to the degenerative process. Results indicate that a higher BMI level could not only increase the degeneration level but also lead to a larger degenerative volume of cartilage. Due to the elevated baseline of inflammation in the obese joint, the relative contributions of inflammation and mechanical damage might vary as cartilage degeneration progressed. This computational framework combines for the first time obesity-associated inflammation and mechanical loading in knee OA. It could be extended by specifying different degenerative pathways in cartilage degeneration. With further calibration, the framework has the potential to empower the identification of different phenotypes and endotypes of OA.

**Supplementary Information:**

The online version contains supplementary material available at 10.1007/s10237-026-02072-8.

## Introduction

Osteoarthritis (OA) is a complex heterogeneous disease that causes major disability, responsible for nearly 2% of total years lived with disability globally (James et al. 2018). In particular, over 80% of global burden of OA are from the knee (James et al. 2018). Approximately 7.6% of the worldwide population was affected by OA in 2020, representing a 132.2% increase since 1990 (Steinmetz et al. 2023). Mechanisms of OA development still remain under investigation, but it is now understood that the progression of OA involves the whole joint and includes multifaceted factors such as genetics, age, gender, injury, tissue metabolism, mechanical loading and inflammation (Tang et al. 2025). These factors interact and contribute to specific phenotypes of OA (Dell’Isola et al. 2016). Since obesity can influence joints both locally and systemically by modulating inflammatory and biomechanical responses (Chang et al. 2018; Collins et al. 2018), it represents a predominant risk factor in the onset and development of OA. In fact, it has been reported that a high BMI accounted for over 20% burden of OA (Steinmetz et al. 2023). Given the rising prevalence of obesity (Blüher 2019) and its substantial contribution to the burden of OA, elucidating the mechanisms of obesity-associated OA is of critical importance.

Excessive joint loading is a critical driving factor of OA due to obesity (Duclos 2016; Chen et al. 2020b), particularly for the knee (MacLean et al. 2016; Stoddart et al. 2021; Joseph et al. 2021; Kim et al. 2021; Li et al. 2022). As the primary weight-bearing joint that supports the upper body, the compressive force in the knee is a direct reflection of changes in body weight. It has been reported that a decrease of one unit in body mass correlated with a four-unit decline in the load of the knee joint (Messier et al. 2005). The increase of the joint load can cause a higher risk of mechanical damage in articular cartilage (Sun 2010; Párraga Quiroga et al. 2017; Riemenschneider et al. 2019; Vazquez et al. 2019), which is prone to the onset and development of knee OA. However, it has been challenging to define and diagnose early knee OA due to its heterogeneous pathological characteristics (Mahmoudian et al. 2021; Emery et al. 2019). The pathology of OA is a consecutive progress from a symptomatic stage to structural erosions (Hawker et al. 2008; Case et al. 2015; Eitner et al. 2017; Nees et al. 2019). At present, two grading systems are commonly used to assess the progression of knee OA, Kellgren-Lawrence (KL) (Kohn et al. 2016) and Osteoarthritis Research Society International (OARSI) atlas criteria (Pritzker et al. 2006; Altman and Gold 2007). They follow the ordinal scales and are unable to well identify the early-stage where radiographic changes of the knee are unobservable. Additionally, a few scoring methods, such as Whole Organ Magnetic Resonance Imaging Score (WORMS), Knee Osteoarthritis Scoring System (KOSS) and MRI Osteoarthritis Knee Score (MOAKS), were developed based on magnetic resonance imaging (MRI) to provide more sensitive assessments on early OA (Roemer et al. 2022). However, an established standard for phenotypic characterisation based on those methods is still lacking.

The degeneration of cartilage is a main hallmark of OA at an early stage, exhibiting compositional and metabolic disruptions (Homandberg et al. 1998; Sokolove and Lepus 2013; Sanchez-Adams et al. 2014; Ismail et al. 2015; Orlowsky and Kraus 2015; Nguyen et al. 2017; Ismail et al. 2017; Nickien et al. 2017; Pérez-García et al. 2019; Peng et al. 2021; Segarra-Queralt et al. 2024). The disruptions include mechanical damage of collagen, the loss of proteoglycans or fixed charge density and activated chronic inflammation (Mukherjee et al. 2020). Tissue softening and the increase of permeability are the primary outcomes of those disruptions in cartilage degeneration. The early variations in the stiffness of cartilage and permeability have been reported prior to detectable structural signs (Song et al. 2024). In particular, obesity is a key regulator responsible for the alterations of the material properties of cartilage (Collins et al. 2018). In addition to the aforementioned biomechanical effects, obesity can also stimulate inflammation in cartilage degeneration. Adipokines are overproduced due to the excess adipose tissue and can disturb the metabolic balance of cartilage turnover (Wang and He 2018; Collins et al. 2021; Zhang et al. 2022). This multi-scale regulation of obesity is a critical factor contributing to the cartilage degeneration in knee OA. However, it remains a challenge to examine the independent effects of obesity due to the complex and interacting pathways of inflammation and biomechanical responses in cartilage degeneration (Seedhom 2006; Messier et al. 2013; Vincent and Wann 2019; Chen et al. 2020a; Reina et al. 2017).

Computational approaches are well suited for integrating diverse pathways of cartilage degeneration across multi-scales. In the context of OA, the knee biomechanics, including the mechanics of articular cartilage, has been commonly explored by using the Finite Element (FE) method (Wilson et al. 2004; Adouni et al. 2012; Klets et al. 2016; Boyd et al. 2016; Sun et al. 2016; Imeni et al. 2020; Al Khatib et al. 2022; Orava et al. 2022; Adouni et al. 2024; Yao et al. 2024; Hu et al. 2024; Cooper et al. 2025). Several computational algorithms have been established to study the development of cartilage degeneration (Hosseini et al. 2014; Stender et al. 2016; Mononen et al. 2016). However, those algorithms are primarily driven by mechanical damage, while biochemical factors are neglected. This limitation also appears to their derivative studies (Klets et al. 2018; Liukkonen et al. 2018; Mononen et al. 2019; Elahi et al. 2023; Orozco et al. 2024). By contrast, numerical models emerge to simulate the metabolic regulations of cartilage degeneration (Baker et al. 2017; Kar et al. 2016; Campbell et al. 2019; Lesage et al. 2022; Segarra-Queralt et al. 2022; Ferrao Blanco et al. 2024; Lai and Lacroix 2025). Since cartilage degeneration is a process that involves the cascade of both biomechanical and inflammatory responses, there are some attempts recently to simulate the mechanobiological mechanisms of cartilage degeneration (Kapitanov et al. 2016; Eskelinen et al. 2020; Segarra-Queralt et al. 2023; Rahman et al. 2023) by coupling mechanical and biochemical factors. Despite difficulties in model validation, integrative computational models contribute to identifying critical pathways and their interactive effects during cartilage degeneration. As aforementioned, obesity plays a predominant role in the multi-scale regulation of the degenerative process of cartilage. However, to date, there is no computational model that integrates the obesity-related coeffects of inflammation and mechanics on the degeneration of cartilage in knee OA. In particular, only one previously established model (Lai and Lacroix 2025), from our prior work, has considered the inflammatory effects of obesity in OA. Previous computational research (Sun et al. 2016; Klets et al. 2018; Liukkonen et al. 2018; Adouni et al. 2024; Orozco et al. 2024) mostly focuses on the biomechanical effects of obesity and could not provide effective means to simulate the cartilage degeneration modulated by obesity-related inflammation and mechanics.

This study aimed to investigate the inflammatory and biomechanical effects of obesity on the cartilage degeneration. A novel multi-scale computational framework was developed, for the first time, integrating the interplay between obesity-associated inflammation and the biomechanics of the knee joint during cartilage degeneration. The sensitivity of the key parameters that govern the degenerative process was analysed within this framework. To evaluate the effects of obesity on cartilage degeneration, BMI levels were varied as the main attribute of the knee joint and the contributions of inflammation and mechanical damage were examined.

## Materials and methods

### Finite element modelling strategy

A validated subject-specific FE model and generic data of the human tibiofemoral joint (Cooper et al. 2023) were used for the biomechanical simulation. The subject-specific data including experimental results, imaging and computational model settings were collected as part of the Institute of Medical and Biological Engineering Knee Dataset (University of Leeds). The experimental implementation was approved by East Midlands - Leicester South Research Ethics Committee (18/EM/0224). The left human cadaveric knee (LTKN8941) was donated by a male of 61-year-old (BMI = 18 kg/m$^2$) without meniscal extrusion.

The subject-specific FE model was modified from this validated hyperelastic model (LTKN8941) (Cooper et al. 2023) in Abaqus 2021 (Dassault Systèmes, Vélizy-Villacoublay, France). The modified subject-specific knee joint model comprises of a total of 73,124 quadratic tetrahedral elements of type C3D10 for the tibia, femur and menisci, as well as 107,446 quadratic tetrahedral elements of type C3D10MP for tibiofemoral cartilages. Tibia and femur were considered as homogeneous materials with isotropic elasticity (Cooper et al. 2025). Femoral and tibial cartilages were modelled as isotropic poroelastic materials (Klets et al. 2016) discretised on an element-wise basis according to the progression of degeneration. The medial and lateral meniscus were assigned with a transverse isotropic property with 50 MPa for the out-of-plane shear modulus and 159.6 MPa for the out-of-plane Young’s modulus (Imeni et al. 2020). To simulate the restricted meniscal movement by the attachment of ligaments, each meniscal horn was respectively connected to tibial plateau by 15 linear spring elements. The sum of spring stiffness was equivalent to the meniscal modulus in the circumferential direction at each side (Yao et al. 2024), and the stiffness of each spring element was 10.64 MPa. Table 1 summarises the material parameters implemented in the FE model at baseline. The sensitivity of material properties is presented in Appendix A, compared with the results from Cooper et al. (2023).

**Table 1:** The baseline parameters of tissue material in the FE model

Material property	Young’s modulus (MPa)	Shear modulus (MPa)	Poisson’s ratio	Void ratio	Permeability (10$^{-3}$ mm$^4$/Ns)
Femur and tibia Cooper et al. (2025)	15,000		0.3		
Meniscus Imeni et al. (2020)	20	7.69	0.3		
	20	50	0.01		
	159.6	50	0.01		
Articular cartilage Klets et al. (2016)	10		0.15	4	1

An axial compressive force, which can be scaled by the body weight to represent the loading at heel strike of a stance phase, was implemented to a control point kinematically coupled to the nodes on the upper surface of the femur. This loading condition was simulated within 0.1s using a transient consolidation analysis, where the fluid exchange was negligible. The surface nodes of cartilage proximal to joint cavity were permeable to ensure stable convergence (Stender et al. 2017). To stabilise the FE model by smoothing the contacts of cartilage-cartilage (CC) and cartilage-meniscus (CM) (Mononen et al. 2019; Cooper et al. 2025), varus-valgus rotation was only allowed when the other degrees of freedom (DoFs) were constrained. Hard surface-to-surface contacts were used for the discretisation method with finite sliding. The friction coefficients were set to 0.1 and 0.15 respectively for CC and CM contacts (Cooper et al. 2025), using the penalty method. The sensitivity of friction coefficients is shown in Appendix A.

**Fig. 1:**
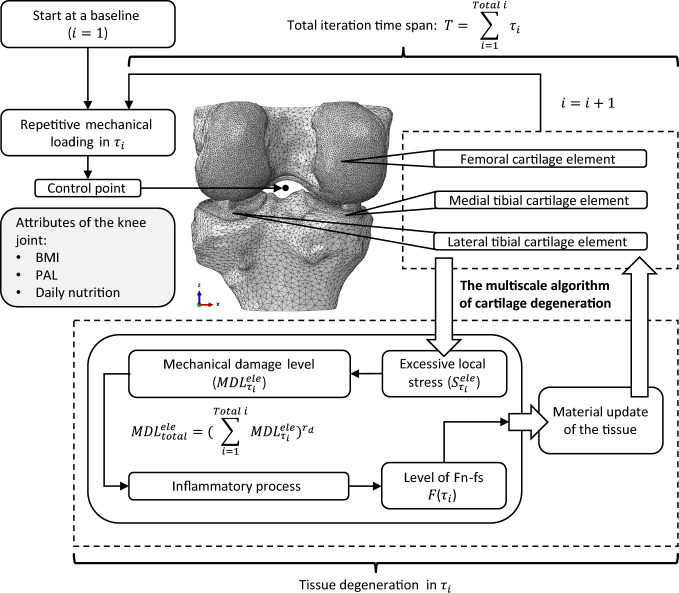
The integrative multi-scale framework of the knee joint degeneration in mechanics and inflammation. Physical activity level (PAL) is defined by the ratio of total daily energy expenditure (TEE) to basal metabolic rate (BMR) (Brooks et al. 2004), and daily nutrition is measured by the ratio of daily calorie intake to BMR. Controlling the values of the two attributes (PAL and daily nutrition) at 1 represents no physical activity and dietary interventions (Lai and Lacroix 2025)

### The multi-scale algorithm of cartilage degeneration

The effect of stress accumulation has been shown to induce cartilage damage, which influences joint mechanics and may accelerate tissue wear. Within this framework, the loading condition was assumed to be repetitive over the time span, resulting in mechanical degeneration. Namely, the cumulative damage to cartilage constituents is attributed to excessive stress from a repeated loading condition within the simulated period. The levels of cartilage mechanical damage were accumulated iteratively by Eqs. (1) and (2) (Mononen et al. 2016).12where *MDL* is the mechanical damage level within the time interval $\tau$ at iteration *i*, *ele* is the label of each cartilage element. *S* is the maximum principal stress of each element and $S_\mathrm{{threshold}}$ is the threshold of maximum principal stress leading to cartilage degradation. $MDL_\mathrm{{total}}^\mathrm{{ele}}$ is the total damage level accumulated from the first iteration due to the inability of healing. In the study by Mononen et al. (2016), the slope of accumulating the mechanical degeneration level could be determined by a root parameter. In this framework, $r_{d}$ measures the accumulation of the total mechanical damage level that contributes to regulating inflammation by the end of each iteration. Due to the lack of evidence on determining both $S_\mathrm{{threshold}}$ and $r_{d}$, they were set to 0.3 MPa and $\dfrac{2}{3}$ at the baseline according to the initial biomechanical response of this FE model.

Since inflammation evolves simultaneously with the tissue damage due to mechanics, a general ODE-based model of adipokine-mediated OA inflammation (Lai and Lacroix 2025) was employed to simulate the cumulative damage, accounting for the combined effects of inflammation and mechanics. $\textit{ode15s}$ in MATLAB (R2022a, The Math Works, Inc., Natick, MA, USA) was used to solve ODEs, where the time span was 1 month for the update of material properties at each iteration. The inflammation model Eqs. (3) to (7) was parameterised Lai and Lacroix (2026) according to the estimation of mediator half-lives (Moise and Friedman 2019; Liu et al. 2021; Rahman et al. 2023; Klein et al. 1996; Homandberg et al. 1998), detailed in Appendix B.

3 $$\begin{aligned} &  \dfrac{dP_c\left( \tilde{\tau _i}\right) }{d\tilde{\tau _i}} = \left[ C_0+ C_1\cdot \dfrac{P_c^n\left( \tilde{\tau _i}\right) }{C_2^n+P_c^n\left( \tilde{\tau _i}\right) }+\right. \nonumber \\ &  \quad C_3\cdot \dfrac{A_d^n\left( \tilde{\tau _i}\right) }{C_4^n+A_d^n\left( \tilde{\tau _i}\right) }+ \left. C_5\cdot \dfrac{F^n\left( \tilde{\tau _i}\right) }{C_6^n+F^n\left( \tilde{\tau _i}\right) } \right] \nonumber \\ &  \quad \cdot \dfrac{C_7^n}{C_7^n+A_c^n\left( \tilde{\tau _i}\right) }- D_1\cdot P_c\left( \tilde{\tau _i}\right) \end{aligned}$$

4 $$\begin{aligned} &  \dfrac{dA_c\left( \tilde{\tau _i}\right) }{d\tilde{\tau _i}} = C_8\cdot \dfrac{P_c^n\left( \tilde{\tau _i}\right) }{C_9^n+P_c^n\left( \tilde{\tau _i}\right) }+ C_{10}\cdot \dfrac{F^n\left( \tilde{\tau _i}\right) }{C_{11}^n+F^n\left( \tilde{\tau _i}\right) } \nonumber \\ &  \quad -D_2\cdot A_c\left( \tilde{\tau _i}\right) \end{aligned}$$

5 $$\begin{aligned} &  \dfrac{dM\left( \tilde{\tau _i}\right) }{d\tilde{\tau _i}} = \left[ C_{12}+ C_{13}\cdot \dfrac{P_c^n\left( \tilde{\tau _i}\right) }{C_{14}^n+P_c^n\left( \tilde{\tau _i}\right) }+ \right. \nonumber \\ &  \quad C_{15}\cdot \dfrac{A_d^n\left( \tilde{\tau _i}\right) }{C_{16}^n+A_d^n\left( \tilde{\tau _i}\right) } \left. \right] \cdot \dfrac{C_{17}^n}{C_{17}^n+A_c^n\left( \tilde{\tau _i}\right) }- D_3\cdot M\left( \tilde{\tau _i}\right) \end{aligned}$$

6 $$\begin{aligned} &  \dfrac{dA_d\left( \tilde{\tau _i}\right) }{d\tilde{\tau _i}} = C_{18}+ \left[ C_{19}\cdot \ \dfrac{BMI^\mathrm{{meas}}}{BMI^\mathrm{{std}}} \right. \nonumber \\ &  \quad \left. \cdot \dfrac{DailyCal}{BMR\cdot PAL} \right] \cdot \dfrac{C_{20}^\mathrm{{nex}}}{C_{20}^\mathrm{{nex}}+A_d^\mathrm{{nex}}\left( \tilde{\tau _i}\right) }- D_4\cdot A_d\left( \tilde{\tau _i}\right) \end{aligned}$$

7 $$\begin{aligned} &  \dfrac{dF\left( \tilde{\tau _i}\right) }{d\tilde{\tau _i}} = C_{21}\cdot M\left( \tilde{\tau _i}\right) +MDL_\mathrm{{total}}^\mathrm{{ele}}-D_5\cdot F\left( \tilde{\tau _i}\right) \end{aligned}$$

where $P_c\left( \tilde{\tau _i}\right)$, $A_c\left( \tilde{\tau _i}\right)$, $M\left( \tilde{\tau _i}\right)$, $A_d\left( \tilde{\tau _i}\right)$ and $F\left( \tilde{\tau _i}\right)$ are the concentrations of pro- and anti-inflammatory cytokines, matrix metalloproteinases (MMPs), adipokines and fibronectin fragments (Fn-fs) at time $\tilde{\tau _i}$. Initial conditions of the inflammatory simulation are the steady state at the first iteration and updated according to the mediator concentrations from $\tau _{i-1}$. Thus far, the connection between the pathways of tissue degradation resulting from inflammation and biomechanics is still unclear in OA. Given the little consensus on the driven mechanism of tissue destruction, cartilage material properties were updated exclusively to local elements exhibiting mechanical damage in this framework, according to the level of inflammation at $\tau _{i}$. In addition, the matrix integrity of undamaged cartilage elements was preserved to keep the consistency of the material property baseline. The aggregate modulus of cartilage decreases due to the loss of structural integrity while permeability increases due to the loss of proteoglycan during OA (Nickien et al. 2017; Peters et al. 2018; Hu et al. 2024; Song et al. 2024). It has been suggested by previous studies (Hosseini et al. 2014; Elahi et al. 2023) that stress- and strain-orientated constituent damage interact and contribute jointly to the degeneration progression. Thus, the mechanical damage to different cartilage constituents and the corresponding changes in the material property were assumed to be synchronised, and Eqs. (8) and (9) were constructed to approximate the element-wise material variations due to cartilage tissue turnover.

8 $$\begin{aligned} E_{\tau _{i+1}}^\mathrm{{ele}} = E_{0}^\mathrm{{ele}}\cdot \left[ 1-\omega _{e}\cdot \left( 1-\dfrac{\Delta F_{\tau _i}^\mathrm{{ele}}}{F_\mathrm{{max}}}\right) \right] \end{aligned}$$

9 $$\begin{aligned} k_{\tau _{i+1}}^\mathrm{{ele}} = k_{0}^\mathrm{{ele}}\cdot \left[ 1+\omega _{k}\cdot \left( 1-\dfrac{\Delta F_{\tau _i}^\mathrm{{ele}}}{F_\mathrm{{max}}}\right) \right] \end{aligned}$$

where $E_{\tau _{i+1}}^\mathrm{{ele}}$ and $k_{\tau _{i+1}}^\mathrm{{ele}}$ are respectively the Young’s modulus of cartilage and the cartilage permeability during iteration $i+1$ after the tissue turnover from the last iteration. To ensure the stabilisation of the FE model, two boundary parameters ($\omega _{e}$ and $\omega _{k}$) set the minimum Young’s modulus and the maximum permeability respectively during tissue degradation. $\omega _{e}$ was set to 0.99 to prevent the convergence issue resulting from the damage concentrated to the local elements that may fail at nearly zero energy (Párraga Quiroga et al. 2017). $\omega _{k}$ was estimated to be 3, ensuring that the maximum permeability is fourfold the baseline during the OA progression (Hu et al. 2024). $\Delta F_{\tau _{i}}^\mathrm{{ele}}$ is the difference between the current and maximum level of Fn-fs for each element, where the maximum level of Fn-fs is denoted by $F_\mathrm{{max}}$. Since fibronectin contributes to the assembly of ECM and the release of Fn-fs is from the disruption of fibronectin (Pérez-García et al. 2019), the level of ECM structural integrity at iteration *i* is demonstrated by a reduction factor ($\eta _{i}^\mathrm{{ele}}$), the ratio of $\Delta F_{\tau _{i}}^\mathrm{{ele}}$ to $F_\mathrm{{max}}$. The degeneration level of each element is correspondingly calculated as $1 - \eta _{i}^\mathrm{{ele}}$, which reflects the adaptation of material properties in the elastic modulus and permeability according to the level of Fn-fs.

**Table 2:** The parameters implemented in the multi-scale algorithm of cartilage degeneration

Parameter	Description	Value	Reference
$S_\mathrm{{threshold}}$ (MPa)	The threshold of maximum principal stress leading to mechanical damage	$\lbrace 0.1, 0.2, 0.3, 0.4, 0.5 \rbrace$	Estimated
$r_d$	The constant that governs the cumulative rate of mechanical damage	$\Bigg \lbrace \dfrac{1}{3}, \dfrac{2}{3}, 1, \dfrac{3}{2}, 2 \Bigg \rbrace$	Estimated
$\omega _{e}$	The boundary of the minimum Young’s modulus for cartilage elements	0.99	Párraga Quiroga et al. (2017)
$\omega _{k}$	The boundary of the maximum permeability for cartilage elements	3	Hu et al. (2024)
$F_\mathrm{{max}}$	The boundary of the maximum level of Fn-fs	$\lbrace 25, 50, 75, 100 \rbrace$	Estimated
$BMI^\mathrm{{meas}}$	The BMI value in the health profile	$\lbrace 18, 25, 36 \rbrace$	Estimated
*PAL*	The constant level of physical activity	1	Lai and Lacroix (2026)
$\dfrac{DailyCal}{BMR}$	The nutritional term defined by the ratio of daily calorie intake to BMR	1	Lai and Lacroix (2026)

At baseline, $S_\mathrm{{threshold}} = 0.3$ MPa, $r_d = \dfrac{2}{3}$, $F_\mathrm{{max}} = 50$

### Integrative multi-scale computational framework

By integrating the obesity-associated inflammation and mechanics to simulate the degeneration of cartilage, a multi-scale computational framework was developed (Fig. 1). During the overall simulation period (*T*), the degeneration level of cartilage tissue was iteratively computed by the multi-scale algorithm of cartilage degeneration that incorporates mechanical and inflammatory regulations. As a critical progressive event in OA, cartilage degeneration involves the pathological changes of different constituents in ECM. The loss of proteoglycan and the disruption of collagen in cartilage ECM are two primary consequences of the interplay between inflammation and mechanics in the pathogenesis of OA (Peng et al. 2021). Since these two pathological changes occur concurrently in the progressive degeneration, it is challenging to examine the contribution of each individual constituent. Computational methods have shown potentials to individually analyse the role of a degenerative pathway in different OA etiologies (Elahi et al. 2023). However, timescales significantly vary in the metabolic mechanisms and damage of different cartilage compositions (Nguyen et al. 2017). Complex time interactions may cause compatible issues in simulating the evolution of OA due to the excessive precision in computing different compositional turnovers. To overcome this when considering the multi-scale effects of obesity, the production of Fn-fs level was modelled to encapsulate the progressive degeneration (Pérez-García et al. 2019), and the update interval for cartilage tissue properties was estimated, ensuring the consistency in the temporal variations of material properties. Since degradation fragments and degeneration are generally on a month-basis (Yue et al. 2021), the minimum time increment at baseline ($\tau _i$) was set to 1 month for each iteration. The sensitivity of $\tau _i$ to the progression of degeneration was shown in Appendix C. Within $\tau _i$, the loading condition was assumed to be repeated by a certain number of cycles leading to mechanical damage. In addition, BMI, physical activity level (PAL) and daily nutrition are three attribute parameters of the knee joint, in relation to the loading condition and the inflammatory activities.

### Computational analysis of cartilage degeneration

**Fig. 2:**
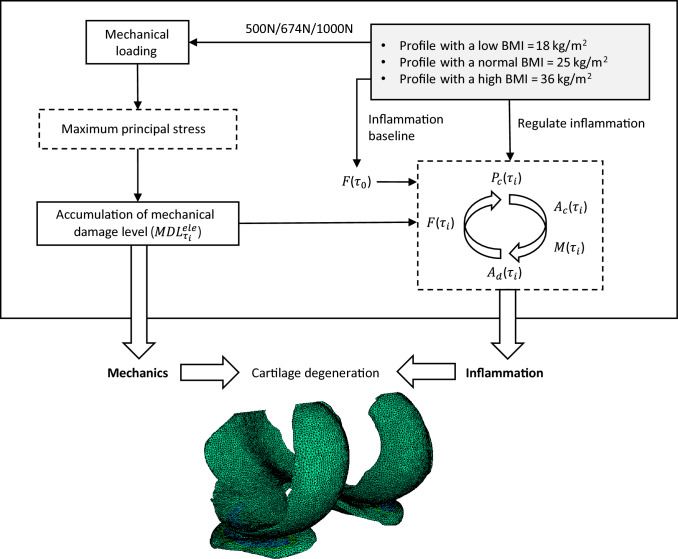
Simulations of cartilage degeneration for three health profiles (low BMI, normal BMI and high BMI)

The sensitivity of two parameters ($r_d$, $F_\mathrm{{max}}$) governing the progressive degeneration of each element in this algorithm was numerically analysed due to the lack of experimental evidence. Synthetic data of maximum principal stress (ranging from 0 to 10 MPa) on individual elements of cartilage was generated for the sensitivity analysis at a single iteration. Table 2 presents the baseline values implemented in the computational framework and the parameters proportionally adjusted within the same order of magnitude for sensitivity analysis. Moreover, to evaluate the variability of cartilage degeneration due to obesity and the heterogeneity of degenerative mechanisms in this framework, three health profiles were generated for the subject-specific FE model when controlling PAL and the nutrition factor to be constant, as illustrated in Fig. 2. The framework presented in Fig. 1 was implemented in MATLAB (R2022a, The Math Works, Inc., Natick, MA, USA), coupled with Abaqus 2021 (Dassault Systèmes, Vélizy-Villacoublay, France), to govern the multi-scale simulations. The variations of biomechanics and inflammatory activities were simulated for 5 years (60 iterations). The estimated value of $S_\mathrm{{threshold}}$ was varied to evaluate the sensitivity of stress threshold for each health profile (Table 2). In addition, the inflammatory and mechanical effects of obesity were examined for three health profiles simulated with baseline parameters.

## Results

### Sensitivity of mechanical damage accumulation and boundary of cartilage integrity

**Fig. 3:**
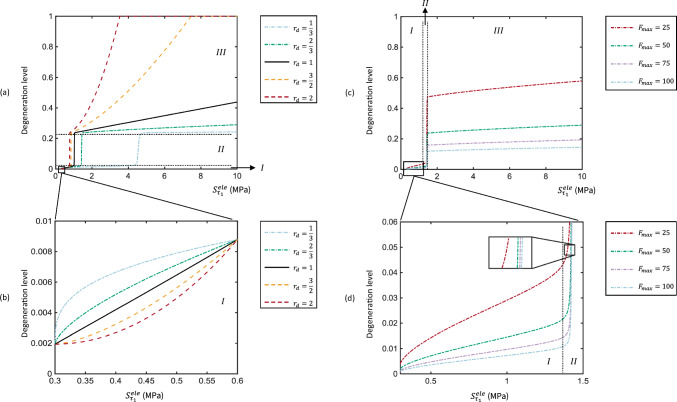
Sensitivity of $r_d$ and $F_\mathrm{{max}}$ to the degenerative process resulted from the accumulation of mechanical damage

The sensitivity of approaches used to govern the cartilage degeneration rate and magnitude in the algorithm was analysed by a parameter variation study. The numerical relationship between two parameters ($r_d$, $F_\mathrm{{max}}$) and the degeneration level of an individual element after one iteration was examined (Fig. 3). The degeneration level ranges from 0 to 1, corresponding to a percentage scale from 0% (nondegeneration) to 100% (full degeneration). Three states were found during the progression of degeneration, as presented in Table 3. The initiation of degeneration occurred when the accumulative mechanical damage was relatively low. The increase of maximum principal stress facilitates the accumulation of mechanical damage from $\textit{I}$ to $\textit{III}$.

4

**Table 3:** States of degenerative process over the accumulation of mechanical damage

Degenerative process	Description
$\text {I}$	Initiation of degeneration
$\text {II}$	Transition state between initiated degeneration and chronic inflammation
$\text {III}$	Degeneration with chronic inflammation

It could be seen that $r_d$ controlled the rate of degeneration (Fig. 3a, b) and $F_\mathrm{{max}}$ dominated the magnitude of degeneration (Fig. 3c, d) in this framework. The linear degenerative progression was simulated at $r_d = 1$, implying that the degeneration level was proportional to the maximum principal stress. As reaching the same degeneration level, the degeneration progressed more rapidly during the initiation of cartilage degeneration ($\textit{I}$) when $r_d$ was decreased (Fig. 3b). At $r_d < 1$, the rate of degeneration gradually slowed in the transition between $\textit{II}$ and $\textit{III}$, whereas an opposite trend was observed at $r_d > 1$. In addition, the increase of $F_\mathrm{{max}}$ only magnified the degeneration level in each state of degeneration, as illustrated in Fig. 3c, d. This resulted in a higher degeneration level in all three states when the accumulation of mechanical damage was identical, suggesting reduced resistance to degeneration at the same stress level.

### Sensitivity of the stress threshold leading to cartilage damage and degeneration

**Fig. 4:**
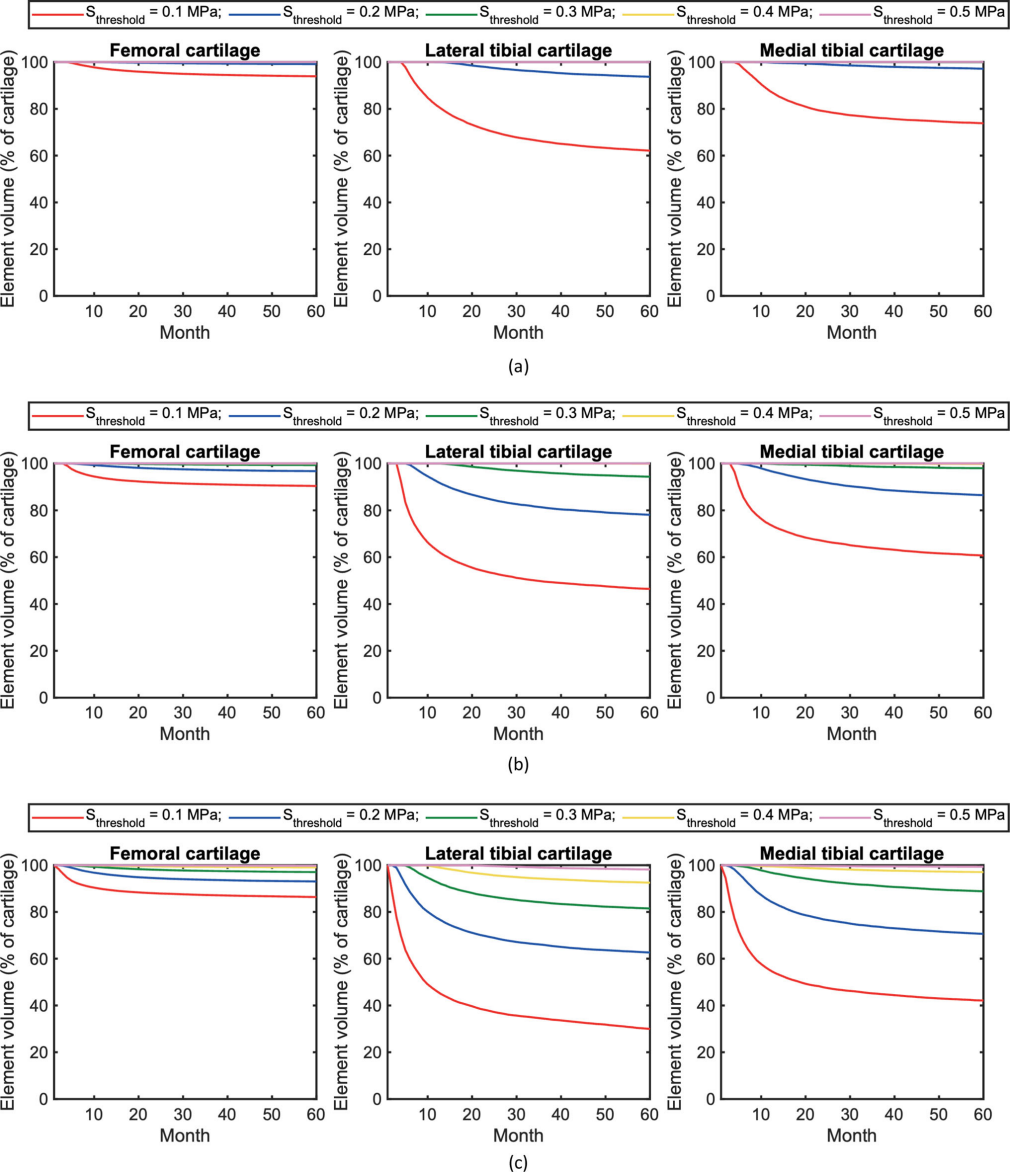
Temporal variations in the percentage of nondegenerative cartilage volume when $S_\mathrm{{threshold}} = \lbrace 0.1, 0.2, 0.3, 0.4, 0.5 \rbrace$ for different BMI levels: **a** low BMI, **b** normal BMI, and **c** high BMI

The degenerative progression of cartilage was evaluated by calculating the volume percentage of nondegenerative elements at each iteration (Fig. 4). For illustration, cartilage elements of which integrity surpasses 75% of the baseline were labelled as nondegenerative elements. Figure 4 shows that the nondegenerative elemental volume nonlinearly declined in the discrete time sequence of simulations. As the cartilage degeneration iteratively progressed, the proportion of degenerative elemental volume to the total volume of each cartilage tended to converge. This qualitative trend remained similar regardless of the difference in the parameterisation of the stress threshold ($S_\mathrm{{threshold}}$) and the health profile ($BMI^\mathrm{{meas}}$). The decrease of the stress threshold significantly accelerated the degradation process and expanded the degenerative cartilage volume. The maximum volume percentages of degenerative cartilage for a low BMI reached nearly up to 49% at $S_\mathrm{{threshold}} = 0.1$ MPa and 7% at $S_\mathrm{{threshold}} = 0.2$ MPa in the lateral tibial cartilage (Fig. 4a). Likewise, the maximum degenerative volume percentages were found in the lateral tibial cartilage for a normal BMI (nearly 57% at $S_\mathrm{{threshold}} = 0.1$ MPa and 22% at $S_\mathrm{{threshold}} = 0.2$ MPa) and a high BMI (around 71% at $S_\mathrm{{threshold}} = 0.1$ MPa and 39% at $S_\mathrm{{threshold}} = 0.2$ MPa), as presented in Fig. 4b, c respectively. In comparison of three health profiles, the profile with a higher BMI was more sensitive to the stress threshold. The maximum degenerative volume percentage of lateral tibial cartilage increased to around 20% at $S_\mathrm{{threshold}} = 0.3$ MPa and 9% at $S_\mathrm{{threshold}} = 0.4$ MPa with a high BMI (Fig. 4c) when only up to 6% degenerative volume of cartilage was found at $S_\mathrm{{threshold}} = 0.3$ MPa in the profile with a normal BMI (Fig. 4b). Additionally, the sensitivity of the stress threshold appeared to differ for femoral and medial tibial cartilages. This might result from the dissimilar biomechanical responses and intact tissue volumes, which are unlikely to change the overall sensitivity pattern.

**Fig. 5:**
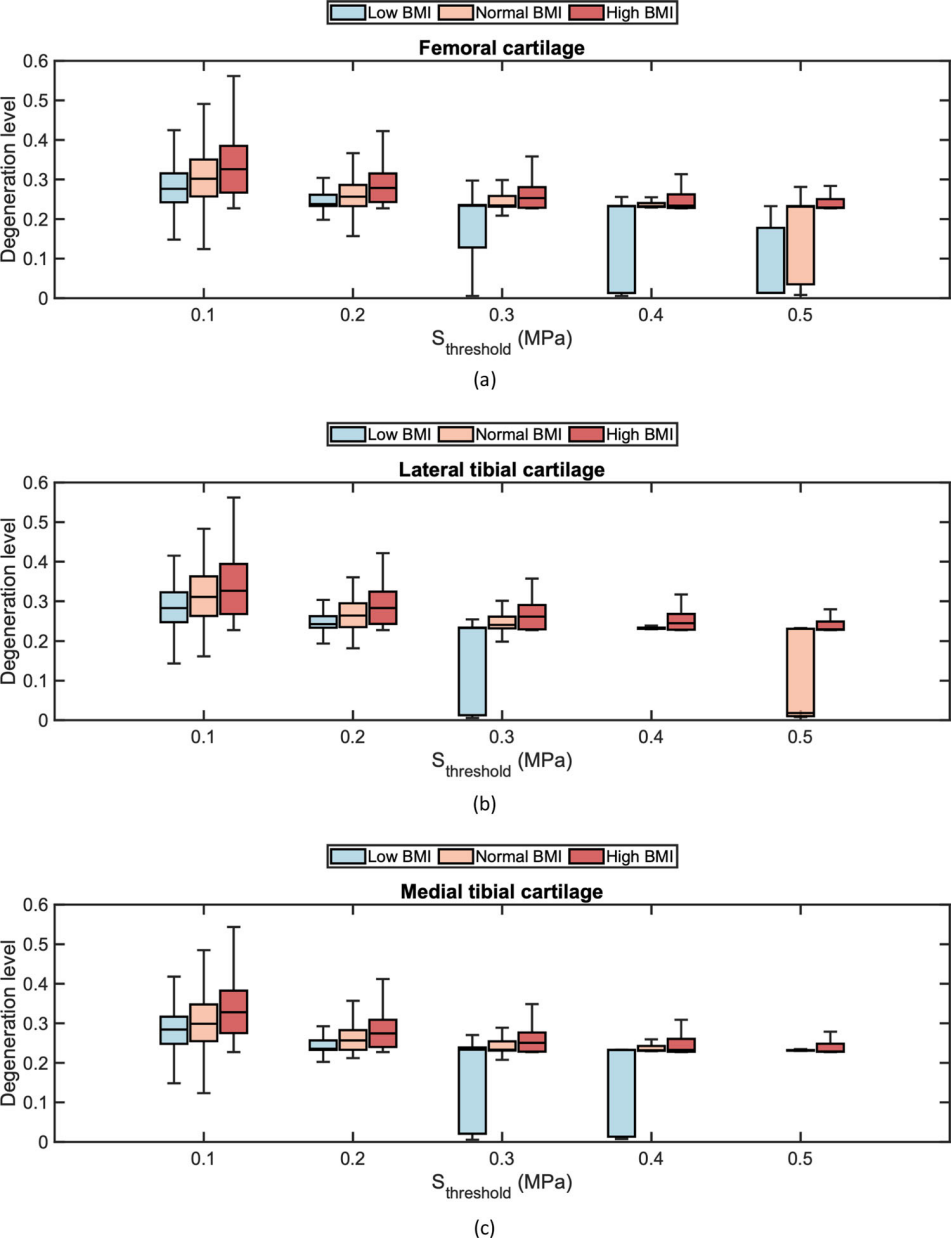
The overall degeneration level of the degenerated elements in **a** femoral cartilage, **b** lateral tibial cartilage and **c** medial tibial cartilage when $S_\mathrm{{threshold}} = \lbrace 0.1, 0.2, 0.3, 0.4, 0.5 \rbrace$ for different BMI levels. In the box chart, the median, interquartile range, and whiskers that extend to the minimum and maximum degeneration levels are indicated

Figure 5 presents the Interquartile Range (IQR) of the degeneration level for degenerated cartilage elements across different health profiles at the end of iterations. It was observed that the median degeneration level of femoral cartilage with a high BMI decreased to 22.9% (IQR 22.8%−25.1%) at $S_\mathrm{{threshold}} = 0.5$ MPa from 32.6% (IQR 26.7%−38.5%) at $S_\mathrm{{threshold}} = 0.1$ MPa (Fig. 5a), indicating that the increase of stress threshold significantly reduces the degeneration. This is likewise to the lateral and medial tibial cartilages for profiles with both a low and normal BMI (Figs. 5b, c). Compared to a high BMI, the health profile with a lower BMI showed a reduced median level of degeneration in both femoral and tibial cartilages regardless of changes in stress threshold. At $S_\mathrm{{threshold}} = 0.1$ MPa, the median degeneration level of the femoral cartilage was 30.2% (IQR 25.7–35.1%) for a normal BMI, decreasing to 27.6% (IQR 24.6–31.6%) in the knee joint with a low BMI (Fig. 5a). It is noteworthy that different locations of cartilage also exhibited variability in the sensitivity of the stress threshold. As the stress threshold was raised from 0.3 MPa to 0.5 MPa, the median degeneration declined from 24.1% (IQR 23.2–26.1%) to 1.8% (IQR 1.1–23.1%) in the lateral tibial cartilage with a normal BMI (Fig. 5b). Similarly, the median degeneration was 23.3% (IQR 23.1–25.4%) at $S_\mathrm{{threshold}} = 0.3$ MPa and 23.1% (IQR 23.1–23.2%) at $S_\mathrm{{threshold}} = 0.5$ MPa in the medial tibial cartilage with a normal BMI (Fig. 5c). Interestingly, the degeneration level of medial cartilage was higher when the threshold was over 0.4 MPa, compared to the lateral cartilage with a low and normal BMI. This is contradictory to the degeneration of tibial cartilages for a high BMI. The significant differences in cartilage compartment were also manifested in the degenerative volume at the identical stress threshold across all three health profiles, suggesting the heterogeneity of cartilage degeneration.

### Effects of obesity-associated inflammation and mechanics during cartilage degeneration

**Fig. 6:**
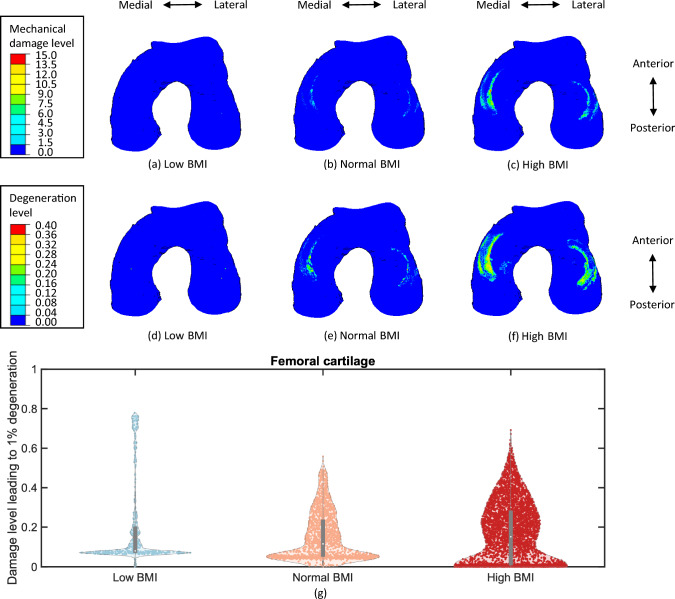
Distribution of mechanical damage level and total degeneration level on the femoral cartilage for a low BMI (**a**) & (**d**), a normal BMI (**b**) & (**e**), and a high BMI (**c**) & (**f**). The damage level leading to 1% degeneration (g) illustrates the relative mechanical and inflammatory contributions to cartilage degeneration. The mechanical damage tends to contribute more with a lower value of this metric compared to inflammation. The width of the KDE curves indicate the density of element distribution. Wider regions correspond to a larger number of cartilage elements, whereas narrower regions represent sparse distributions

**Fig. 7:**
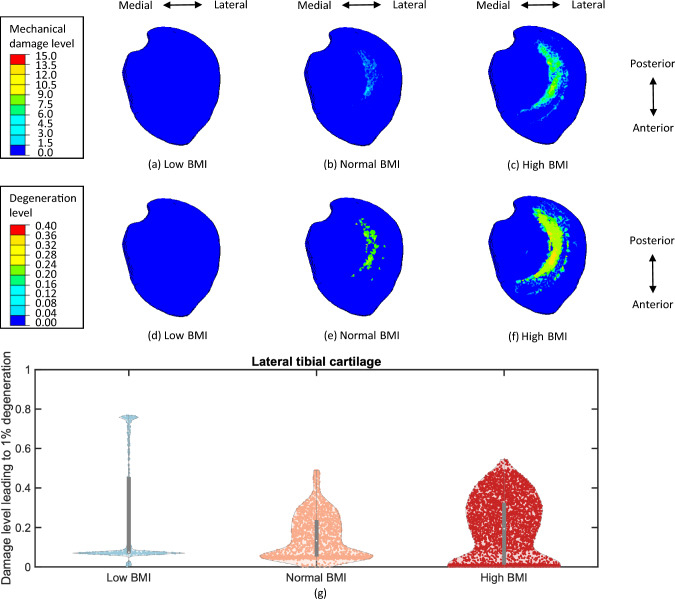
Distribution of mechanical damage level and total degeneration level on the lateral tibial cartilage for a low BMI (**a**) & (**d**), a normal BMI (**b**) & (**e**), and a high BMI (**c**) & (**f**). The damage level leading to 1% degeneration (g) illustrates the relative mechanical and inflammatory contributions to cartilage degeneration. The mechanical damage tends to contribute more with a lower value of this metric compared to inflammation. The width of the KDE curves indicates the density of element distribution. Wider regions correspond to a larger number of cartilage elements, whereas narrower regions represent sparse distributions

**Fig. 8:**
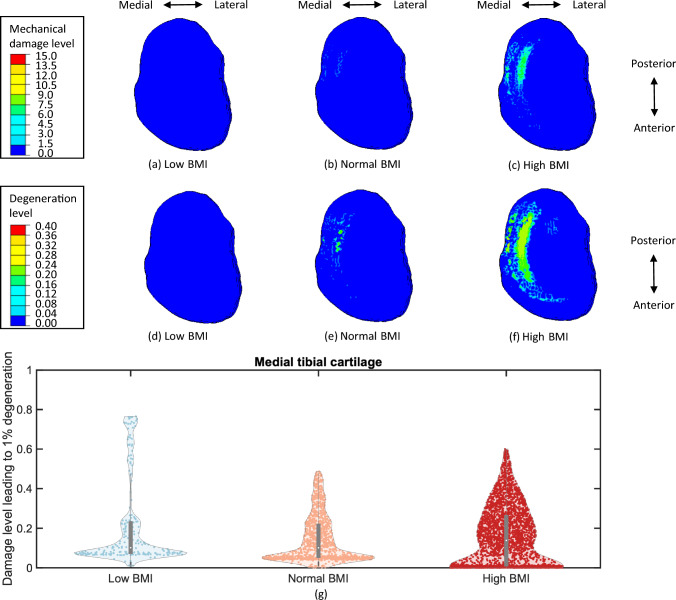
Distribution of mechanical damage level and total degeneration level on the medial tibial cartilage for a low BMI (**a**) & (**d**), a normal BMI (**b**) & (**e**), and a high BMI (**c**) & (**f**). The damage level leading to 1% degeneration (g) illustrates the relative mechanical and inflammatory contributions to cartilage degeneration. The mechanical damage tends to contribute more with a lower value of this metric compared to inflammation. The width of the KDE curves indicate the density of element distribution. Wider regions correspond to a larger number of cartilage elements, whereas narrower regions represent sparse distributions

After five-year simulations of cartilage degeneration, both the cumulative mechanical damage level and the total degeneration level were elevated for the femoral and tibial cartilages with a normal and high BMI in comparison to a low BMI (Figs. 6 to 8). The distributions of mechanical damage and total degeneration differed between femoral and tibial cartilages across the medial and lateral compartments. This variability was more pronounced in the profile with a high BMI, since the increase of BMI resulted in a larger degenerative area of cartilage. Due to the function of menisci, mechanical damage and degeneration appeared along the medial and lateral contact margins of the femoral cartilage (Figs. 6c, f). However, the degenerative region was concentrated laterally on the lateral tibial cartilage (Fig. 7f), and a larger area in degeneration was found compared to the medial tibial cartilage (Fig. 8f), where the degenerative elements were mainly located on the medial side. This was also found in the distribution of mechanical damage (Figs. 7c and 8c), with the medial tibial cartilage showing a less damaged area than the lateral tibial cartilage. It is noteworthy that the distributions of mechanical damage and total degeneration were not matched for the femoral (Figs. 6c and 6f), lateral tibial (Figs. 7c and 7f) and medial tibial cartilage (Figs. 8c and 8f) due to inflammation.

To visualise the contribution of mechanical damage for each cartilage element, the ratio of damage level and total degeneration level was calculated as a metric, representing the damage level that introduces 1% degeneration. Figures 6g, 7g, 8g show the IQR and kernel density estimate (KDE) of the data on degenerative elements with different proportional contributions of mechanics and inflammation. The median value of the mechanical damage level leading to 1% degeneration and the number of degenerative elements were increased with a higher BMI due to the difference in the progression of cartilage degeneration. Accordingly, the median values might not be directly comparable across different health profiles. Instead, the vertically oriented KDE curves illustrated that a wider section occurred at the lower values of this metric from a low BMI to a high BMI. This suggests that the increase in BMI leads to more pronounced inflammatory effects on cartilage degeneration. In addition, the KDE curves displayed an uneven pattern with two peaks of the curve width for three levels of BMI. Particularly, the KDE curves extended upwards, showing the distribution of cartilage elements with larger mechanical contributions to degeneration. At a low BMI, the width of the KDE curve increased forming a first peak, followed by a long decline. A second peak appeared towards the end where the metric reached its highest value near 0.8. Differently, the second peak was found in the middle of the KDE curves for a normal and high BMI. These non-uniform patterns indicate that the contributions of mechanical damage and inflammation are distributed differently across cartilage elements, which may result from the varying states in the degenerative process.

## Discussion

A novel multi-scale framework was developed, integrating for the first time the obesity-associated inflammation and mechanics in the context of knee OA. The complex interplay between inflammatory activities and biomechanical behaviours affected by obesity was simulated by coupling the adipokine-mediated inflammation (Lai and Lacroix 2025) with knee joint biomechanics at both spatial and temporal scales. This framework focused on the cartilage degeneration initiated by accumulative mechanical damage at different levels of inflammation, providing an advanced tool to assess the risk of OA according to the subject-specific joint profiles. In the degeneration algorithm, the heterogeneity of tissue turnover was approached through element-wise simulations of the poroelastic cartilage. The sensitivity of two governing parameters ($r_d$, $F_\mathrm{{max}}$) was studied. Adjusting $r_d$ and $F_\mathrm{{max}}$ could modulate the rate and magnitude of degeneration during progression, respectively. Moreover, the cartilage degeneration was simulated within 5 years (60 iterations) for the subject-specific knee joint (LTKN8964). The degeneration was found to be sensitive to the stress threshold as well as the level of BMI, with higher levels of BMI potentially accelerating the time-varying process. The distribution of mechanical damage and total degeneration was location-specific in cartilages, and obesity considerably elevated their levels. In particular, excess adiposity raised the baseline level of inflammation in cartilaginous environment. Consequently, inflammation became the dominant factor of cartilage degeneration as BMI increased.

Cartilage degeneration is a nonlinear progression during OA due to complex inflammatory and biomechanical regulations (Sanchez-Adams et al. 2014; Peng et al. 2021; Segarra-Queralt et al. 2024). At an early stage, the degeneration is not observable from radiographic assessments, and prodromal symptoms are the critical sign of knee OA (Case et al. 2015; Mahmoudian et al. 2021). Knee pain is an established symptom (Hawker et al. 2008; Emery et al. 2019) whereas it could be the outcome of other symptoms such as inflammatory activities (Nees et al. 2019) and tissue damage (Eitner et al. 2017). As degeneration progresses, the balance of anabolic and catabolic activities is disrupted within cartilage. This leads to chronic inflammation and accumulative mechanical damage accompanied by intermittent pain, ultimately resulting in structural changes. The algorithm of cartilage degeneration in this framework was able to capture three primary hallmarks ($\textit{I}$, $\textit{II}$, $\textit{III}$) for localised degenerative elements when considering the interactions of inflammation and mechanical damage. The repetitive loading over the threshold leads to damage accumulation in articular cartilage (Seedhom 2006; Riemenschneider et al. 2019). This accumulation gradually alters the metabolism of cartilage to initiate degeneration, which corresponds to $\textit{I}$. The damage level accumulates to degrade tissue, and PRRs and DAMPs are activated in the innate immune response (Orlowsky and Kraus 2015), resulting in the transition of anabolism towards catabolism ($\textit{II}$). Progressively, chronic inflammation evolves in the degenerative process (Sokolove and Lepus 2013), corresponding to $\textit{III}$. Nevertheless, there is yet little evidence on the quantitative relationship between the level of accumulative damage and those three states. Degeneration results from the dysregulation of cartilage metabolism that is affected by local and systemic inflammation (Chang et al. 2018; Collins et al. 2021) and crosstalks between surrounding tissues (Findlay and Kuliwaba 2016; Zeng et al. 2020), while individual differences also provide a large variability due to effects of genetics, age, gender and lifestyles (Seedhom 2006). Thus, it is significantly challenging to fully validate the algorithm of cartilage degeneration in this framework. Despite that, two parameters ($r_d$, $F_\mathrm{{max}}$) of the algorithm were able to govern the rate and level of cartilage degeneration, which can minimally represent the outcomes of unmeasurable local and systemic confounding effects rather than extensively simulating them. The degeneration of cartilage has been described through variations in tissue constituents in previous computational models (Hosseini et al. 2014; Stender et al. 2016; Mononen et al. 2016; Párraga Quiroga et al. 2017; Orozco et al. 2018; Mononen et al. 2018; Eskelinen et al. 2020; Rahman et al. 2023; Elahi et al. 2023). In particular, the biomechanical characterisations of tissue material are functions of compositional changes, such as collagen destruction, proteoglycan depletion or the loss of fixed charge density. Loading rate and magnitude (Sun 2010; Párraga Quiroga et al. 2017) are tightly associated with the degradation of different constituents, and its cyclic exposure (Riemenschneider et al. 2019; Vazquez et al. 2019) determines the fatigue life of cartilage material. Thus, results are not able to be quantitatively compared due to the difference in time dependencies and loading conditions. However, tuning $r_d$ could mimic the variations of the degeneration rate that was found in the study by Mononen et al. (2016), which demonstrates an excellent adaptivity of this multi-scale framework. Additionally, the decrease of $F_\mathrm{{max}}$ significantly reduced the cartilage degeneration level at the same stress magnitude. As a product of ECM degradation, the level of Fn-fs is an indication of cartilage degeneration (Homandberg et al. 1998; Pérez-García et al. 2019). The boundary of Fn-fs level could be adapted to individuals with different susceptibilities to cartilage degeneration (Vincent and Wann 2019; Seedhom 2006). In future work, incorporating additional patient-specific data could enable the estimation and interaction analysis of these parameters to characterise different patient groups based on longitudinal assessments of cartilage degeneration. This may contribute to subject-specific simulations of cartilage degeneration, thereby improving patient stratification and investigating personalised treatment strategies.

The stress threshold that determines damage accumulation was found markedly sensitive to the degenerative process. The cartilage degeneration was accelerated and exacerbated with a lower threshold but tended to slow over time. This is consistent with previous models (Mononen et al. 2016; Klets et al. 2018), though the values of threshold are not comparable. In addition to time dependencies and loading conditions, cartilage conditioning (Seedhom 2006), modulated by individual differences, may also underlie the specificity of the threshold. In particular, Vincent and Wann (2019) suggested that the threshold may be decreased if cartilage loses its mechanoprotective mechanisms where transforming growth factor-$\beta$-activated kinase 1 (TAK1) (Ismail et al. 2015) may play a critical role as an important upstream inflammatory regulator in response to mechanical damage. The activation of TAK1 still remains unclear and may not involve soluble mediators (Ismail et al. 2017). Therefore, the damage threshold may vary widely across individuals. Different modelling strategies of tissue damage can also contribute to the uncertainty of the threshold, highlighting the importance of analysing its sensitivity. In this framework, the sensitivity of the threshold is the result of the synergistic interaction of the increased inflammation baseline (Collins et al. 2021) and mechanical loading (Chen et al. 2020b). The results showed that the degeneration was more sensitive to the threshold at a higher level of BMI, while obesity increased the level and volume of degenerative elements. This relationship between obesity and cartilage degeneration is in line with the results from previous computational models (Boyd et al. 2016; Sun et al. 2016; Klets et al. 2018; Liukkonen et al. 2018; Al Khatib et al. 2022; Adouni et al. 2024; Orozco et al. 2024) whereas they have focused on the obesity-mediated biomechanics in knee OA. From a biomechanical perspective, obesity can increase the adduction moment of the knee joint (Kim et al. 2021) and may result in the medial concentration of cartilage contact (Li et al. 2022). In fact, Stoddart et al. (2021) reported a higher risk of OA in the medial compartment of the tibiofemoral joint. However, this study showed that the degeneration of all three profiles appeared to be more severe in the lateral tibial cartilage compared to the medial tibial cartilage. It should be noted that the validated FE model in this framework was created from the healthy knee joint of a male with BMI = 18 kg/m$^2$. The increased risk to the medial compartment might result from the malalignment or muscle weakness caused by a long-term effect of obesity (Chen et al. 2020b). In addition, subject-specific gait pattern (Adouni et al. 2012; MacLean et al. 2016; Adouni et al. 2024) and joint geometry (Boyd et al. 2016; Orozco et al. 2024; Cooper et al. 2025) are tightly associated with the mechanical responses of the knee in OA. In this computational model, the gait kinetics and kinematics of the knee joint were simplified to one loading condition for the study of the interacting effects of obesity-associated mechanical damage and inflammation. Consequently, the effect of the external adduction moment (Adouni et al. 2012) was not included, and compartmental load distribution only resulted from the interactions among joint geometry, contact conformity, and material behaviour. This may explain the higher level of degeneration on the lateral side in this study. In fact, it has been reported that obese individuals may tend to walk at a lower speed so that the knee adduction moment increases (MacLean et al. 2016). However, the major influence of the external adduction moment generally appears across most of the stance phase apart from the heel strike and the first 5% of the gait cycle (Adouni et al. 2012). This study thereby provides conservative estimates of cartilage degeneration under one loading condition resulting from obesity rather than precise predictions. Integrating kinetic and kinematic data of the joint can improve the framework to capture the compartmental degeneration distribution for clinical predictions in the future.

As the first computational framework considering both inflammatory and mechanical effects of obesity, it simulated the progression of mechanical damage and degeneration triggered by the local maximum principal stress of cartilage over a threshold. It was revealed that obesity significantly exacerbated cartilage degeneration which was location-specific according to the mechanical damage. This location specificity is consistent with the computational predictions in the study by Klets et al. (2018) regarding the location of cartilage degeneration due to the effects of body weight. However, the distribution of cartilage degeneration was not congruent with the mechanical damage, a discrepancy that became more pronounced as the BMI level increased. This could be explained by the difference in the contributions of inflammation and mechanical damage due to obesity. The results showed that a higher level of BMI increased the degenerative cartilage volume dominated by inflammation. The previous dynamics analysis (Lai and Lacroix 2025) of obesity-associated inflammation has suggested that obesity can significantly increase the susceptibility of OA inflammation. Moreover, the pivotal role of obesity in OA inflammation has been widely reported (Zhang et al. 2022; Collins et al. 2021; Chang et al. 2018), which is in line with the aforementioned effects of obesity in this study. As degeneration progressed, it was predicted that the accumulation of mechanical damage led to different patterns of degeneration and the contribution of mechanical damage varied across cartilage elements. This suggests that the orientation of local cartilage degeneration may shift with BMI levels between the damage-driven progression and the inflammation-dominated process at an early stage of the obesity-associated OA. However, the relative contributions of mechanical damage against inflammation likely depend on a complex dynamic interplay of individual factors that are not yet fully understood. In addition, the relationship between obesity and numerical variations of cartilage constituents remains unestablished in cartilage degeneration. Accordingly, this framework postulated the synchronised degradation of different compositions (Hosseini et al. 2014; Elahi et al. 2023), which was implicitly approached by the production of Fn-fs. Despite that, the results showed that the average level of cartilage degeneration was positively correlated with BMI, which aligns with the cartilage property alterations due to obesity reported by Collins et al. (2018).

Since BMI level, cartilage metabolisms and loading conditions of the knee joint all vary simultaneously and dynamically according to physical activities and diet (Messier et al. 2013; Collins et al. 2018; Joseph et al. 2021), it is challenging to assess the dynamic evolution of specific obesity effects in OA. Thus, the development of this framework necessitates appropriate simplifications with a set of underlying assumptions. Due to the lack of longitudinal subject-specific data, the validity of this framework was evaluated by qualitatively comparing the degenerative process and the outcomes of obesity with previous findings. The subject-specificity could be improved by including more detailed data such as levels of specific biomarkers, gait patterns and body weight to examine the cartilage degeneration influenced by obesity. In addition, the obesity-mediated inflammatory process was simulated by an ODE-based model of five general mediator groups. Specifying the regulatory network could contribute to a more precise assessment on the mechanism of cartilage degeneration due to obesity (Lai and Lacroix 2025). Similarly, implementing the depth-dependent fibril-reinforced biphasic constitutive model of cartilage (Wilson et al. 2004; Mononen et al. 2018) can also increase the precision by providing more accurate biomechanical behaviours during simulations. Nevertheless, a more granular simulation requires a much more precise patient-specificity of the cartilage material properties. The constitutive model of isotropic poroelasticity could not simulate directional biomechanical responses of cartilage and might underestimate the tensile stress on the contact surface (Klets et al. 2016). However, the primary purpose of this study was to investigate the interactions of obesity-associated mechanical damage and inflammation, thereby the degeneration in this study was a conservative estimate. This framework includes a relatively minimal number of parameters and variables to integrate the effects of obesity on the cartilage degeneration. Aside from the calibration of parameters, the estimation of initial conditions for the knee joint is also limited. Specifically, the initial bone properties were not adjusted according to the changes in BMI. BMI and mechanical properties of subchondral bone were found correlated but still remain disputed (Chen et al. 2020a; Reina et al. 2017). However, an FE study by Orava et al. (2022) indicated that subchondral bone variations had no significant effect on the mechanics of articular cartilage in early OA. Given that, the observed effects of obesity on cartilage degeneration remain valid within the scope of the present study. In the future, this framework could be extended to simulate and predict the risk of cartilage degeneration due to obesity, by including more granular subject-specific data and specifying additional regulatory pathways of OA.

## Conclusion

A novel integrative multi-scale modelling framework was developed to evaluate the inflammatory and biomechanical effects of obesity on cartilage degeneration in knee OA. This is the first computational framework incorporating the degenerative pathway of obesity-associated inflammation. Results suggested that the inflammatory process modulated by BMI could differentiate the progression of cartilage degeneration. Increasing BMI resulted in a higher level of inflammation and stress in cartilage. This altered the threshold of cartilage to mechanical damage and its degenerative process. In the obese profile, a larger degenerative volume of cartilage was found with inflammation contributing more to the cartilage degeneration than mechanical damage. These computational results provide a transparent pathway of cartilage degeneration modulated by obesity. With future calibration, this framework could facilitate the identification of endotypes underlying obesity-related OA.

## Supplementary Information

Below is the link to the electronic supplementary material.

Supplementary file 1 (pdf 157 KB)

Supplementary file 2 (pdf 100 KB)

Supplementary file 3 (pdf 366 KB)

## Data Availability

The core source code of the framework and ongoing updates are available on GitHub at https://github.com/JuntongLai/MCD-OA.git. A clean, archived version of the core source code used in this study can be accessed in the approved repository licenced by GPL3.0 as follows: Lai, Juntong; Lacroix, Damien (2026). Core source code to supplement the manuscript titled “A novel integrative multi-scale framework of inflammation and mechanical loading in knee osteoarthritis”. The University of Sheffield. Software. https://doi.org/10.15131/shef.data.32030283.v1.
