# Supplementary material for: A novel integrative multi-scale framework of inflammation and mechanical loading in knee osteoarthritis
Source: Biomech Model Mechanobiol. 2026 Jun 3;25(3):55. doi: 10.1007/s10237-026-02072-8 (PMC13233665; doi:10.1007/s10237-026-02072-8)
Supplement: Supplementary file 1 — (pdf 157 KB) [file 10237_2026_2072_MOESM1_ESM.pdf]

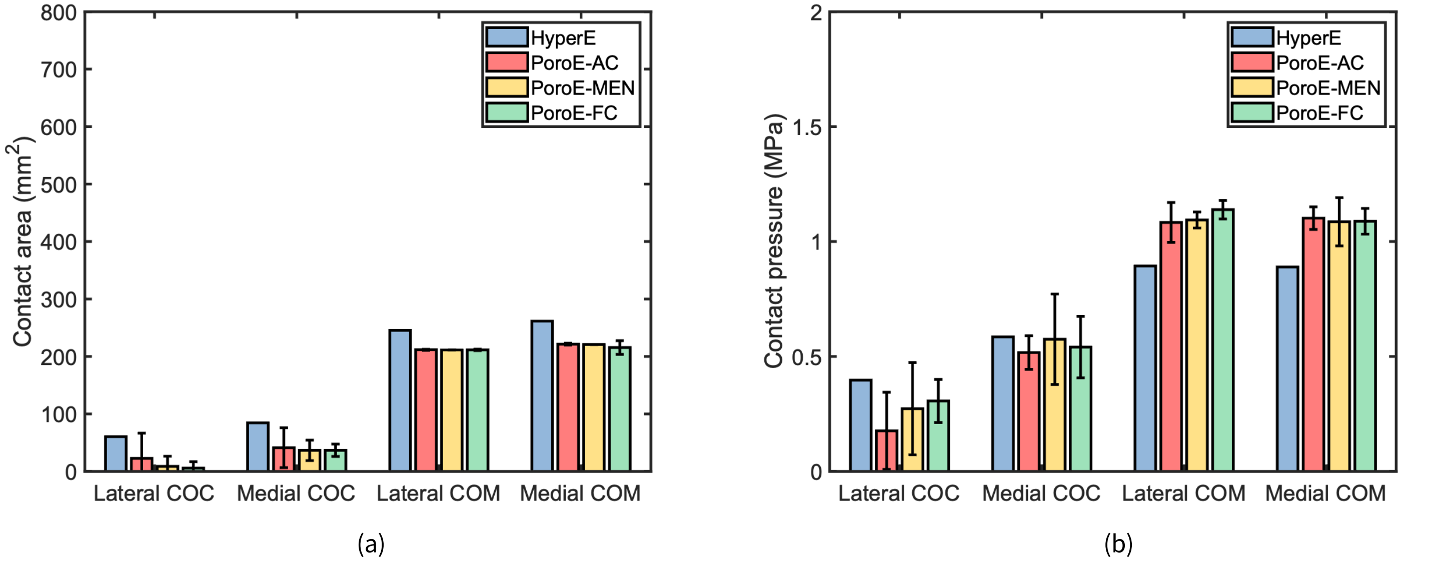

Model cohort	Articular cartilage	Meniscus	Contact friction coefficient
PoroE-AC	[25%, 175%] of baseline properties	Baseline	Baseline
PoroE-MEN	Baseline	[25%, 175%] of baseline properties	Baseline
PoroE-FC	Baseline	Baseline	[25%, 175%] of baseline properties
HyperE	Neo-hookean: C_{10} = 1.0274 D_1 = 0.08	$E_1 = E_2$ = 6 MPa; E_3 = 21 MPa; $v_{in\text{-}plane}$ = 0.3; $v_{out\text{-}plane}$ = 0.2; $G_{out\text{-}plane}$ = 8.08 MPa	$F_{CART\text{-}CART}$ = 0.1 $F_{CART\text{-}MEN}$ = 0.15
