# Supplementary material for: A novel integrative multi-scale framework of inflammation and mechanical loading in knee osteoarthritis
Source: Biomech Model Mechanobiol. 2026 Jun 3;25(3):55. doi: 10.1007/s10237-026-02072-8 (PMC13233665; doi:10.1007/s10237-026-02072-8)
Supplement: Supplementary file 2 — (pdf 100 KB) [file 10237_2026_2072_MOESM2_ESM.pdf]

Appendix B Table. The parameterisation of the obesity-associated inflammation model.

Parameter	Description	Value
C_0	Natural production rate of PICs	0.05
C_1	Stimulated production rate of PICs by PICs	50
C_2	Saturation constant at which the capability of stimulating PIC production signalled by PICs is half of maximum	5
C_3	Stimulated production rate of PICs by adipokines	50
C_4	Saturation constant at which the capability of stimulating PIC production signalled by adipokines is half of maximum	5
C_5	Stimulated production rate of PICs by Fn-fs	50
C_6	Saturation constant at which the capability of stimulating PIC production signalled by Fn-fs is half of maximum	5
C_7	Saturation constant at which the capability of inhibiting PIC production signalled by AICs is half of maximum	5
C_8	Stimulated production rate of AICs by PICs	1×1.5^4
C_9	Saturation constant at which the capability of stimulating AIC production signalled by PICs is half of maximum	1×1.5^3
C_{10}	Stimulated production rate of AICs by Fn-fs	1×1.5^4
C_{11}	Saturation constant at which the capability of stimulating AIC production signalled by Fn-fs is half of maximum	1×1.5^3
C_{12}	Natural production rate of MMPs	0.05
C_{13}	Stimulated production rate of MMPs by PICs	50
C_{14}	Saturation constant at which the capability of stimulating MMP production signalled by PICs is half of maximum	5
C_{15}	Stimulated production rate of MMPs by adipokines	50

Continued on next page

(Continued)

C_{16}	Saturation constant at which the capability of stimulating MMP production signalled by adipokines is half of maximum	5
C_{17}	Saturation constant at which the capability of inhibiting MMP production signalled by AICs is half of maximum	5
C_{18}	The background production rate of adipokines due to the number of adipocytes	500
C_{19}	The background production rate of adipokines due to the size of adipocytes	500
BMI^{std}	The standard BMI	25
C_{20}	Saturation constant at which the capability of reducing adiposity through physical activity is half of maximum	Depending on PAL
C_{21}	Stimulated production rate of Fn-fs by MMPs	3
D_1	Clearance rate of PICs	5.2
D_2	Clearance rate of AICs	1.5×10^3
D_3	Clearance rate of MMPs	4.2
D_4	Clearance rate of adipokines	1.2×10^3
D_5	Clearance rate of Fn-fs	3
nex	The coefficient that governs the nonlinearity of physical activity effects at different BMI levels	Depending on BMI^{meas}
n	Hill coefficient	2
