# Supplementary figures and images for: A novel integrative multi-scale framework of inflammation and mechanical loading in knee osteoarthritis
Source: Biomech Model Mechanobiol. 2026 Jun 3;25(3):55. doi: 10.1007/s10237-026-02072-8 (PMC13233665; doi:10.1007/s10237-026-02072-8)

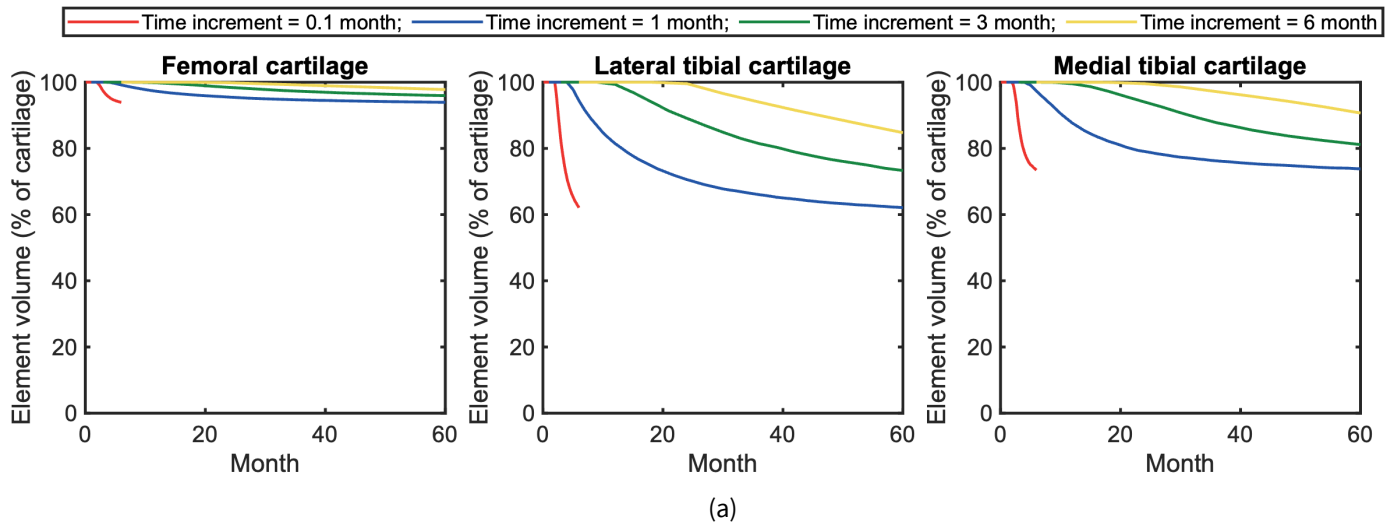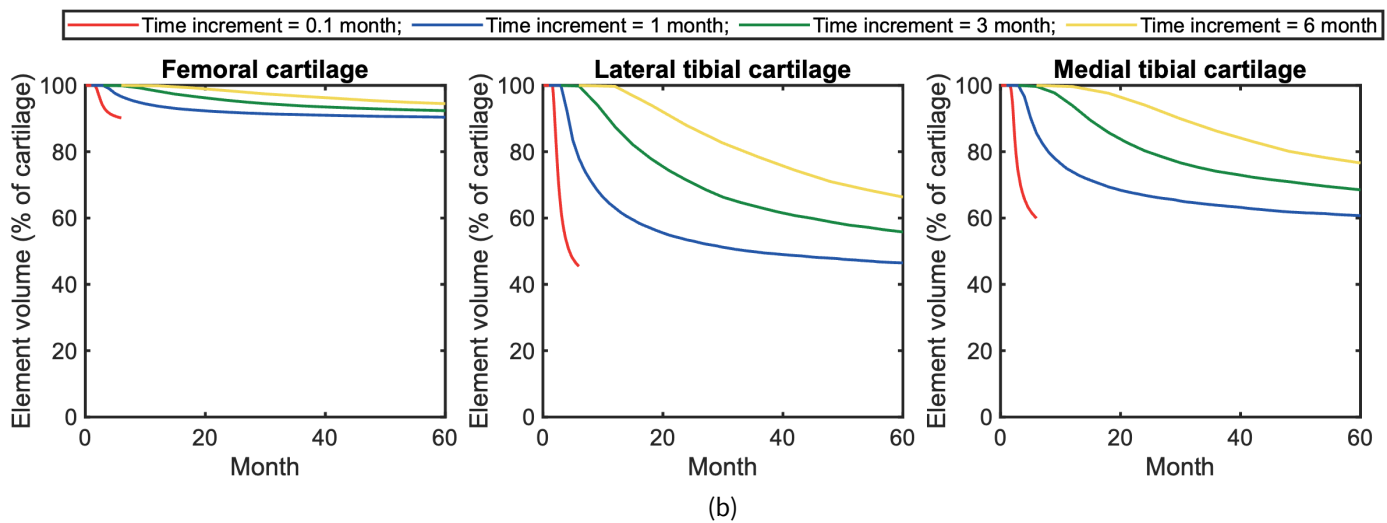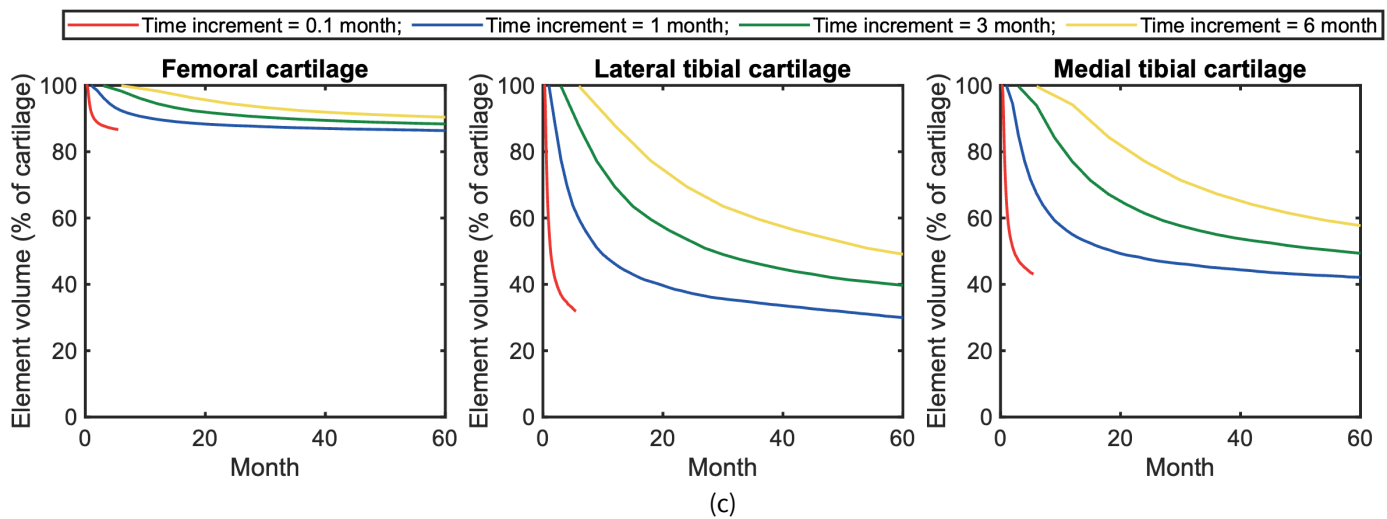

Supplement: Supplementary file 3 — (pdf 366 KB) [file 10237_2026_2072_MOESM3_ESM.pdf]
